# *Growth Arrest-Specific Transcript 5* Associated snoRNA Levels Are Related to p53 Expression and DNA Damage in Colorectal Cancer

**DOI:** 10.1371/journal.pone.0098561

**Published:** 2014-06-13

**Authors:** Jonathan Krell, Adam E. Frampton, Reza Mirnezami, Victoria Harding, Alex De Giorgio, Laura Roca Alonso, Patrizia Cohen, Silvia Ottaviani, Teresa Colombo, Jimmy Jacob, Loredana Pellegrino, Gordon Buchanan, Justin Stebbing, Leandro Castellano

**Affiliations:** 1 Division of Cancer, Department of Surgery and Cancer, Imperial College, Hammersmith Hospital, London, United Kingdom; 2 HPB Surgical Unit, Department of Surgery and Cancer, Imperial College, Hammersmith Hospital, London, United Kingdom; 3 Department of Surgery and Cancer, Imperial College London, St. Mary's Hospital Campus, London, United Kingdom; 4 Department of Histopathology, Hammersmith Hospital, London, United Kingdom; 5 Institute for Computing Applications “Mauro Picone”, National Research Council, Rome, Italy; 6 Colorectal Surgical Unit, Imperial College Healthcare NHS Trust, Charing Cross Hospital, London, United Kingdom; 7 Department of Oncology, Imperial College Healthcare NHS Trust, Charing Cross Hospital, London, United Kingdom; University of Torino, Italy

## Abstract

**Background:**

The *growth arrest-specific transcript 5 gene* (*GAS5*) encodes a long noncoding RNA (lncRNA) and hosts a number of small nucleolar RNAs (snoRNAs) that have recently been implicated in multiple cellular processes and cancer. Here, we investigate the relationship between DNA damage, p53, and the *GAS5* snoRNAs to gain further insight into the potential role of this locus in cell survival and oncogenesis both *in vivo* and *in vitro*.

**Methods:**

We used quantitative techniques to analyse the effect of DNA damage on *GAS5* snoRNA expression and to assess the relationship between p53 and the *GAS5* snoRNAs in cancer cell lines and in normal, pre-malignant, and malignant human colorectal tissue and used biological techniques to suggest potential roles for these snoRNAs in the DNA damage response.

**Results:**

*GAS5*-derived snoRNA expression was induced by DNA damage in a p53-dependent manner in colorectal cancer cell lines and their levels were not affected by DICER. Furthermore, p53 levels strongly correlated with *GAS5*-derived snoRNA expression in colorectal tissue.

**Conclusions:**

In aggregate, these data suggest that the *GAS5*-derived snoRNAs are under control of p53 and that they have an important role in mediating the p53 response to DNA damage, which may not relate to their function in the ribosome. We suggest that these snoRNAs are not processed by DICER to form smaller snoRNA-derived RNAs with microRNA (miRNA)-like functions, but their precise role requires further evaluation. Furthermore, since GAS5 host snoRNAs are often used as endogenous controls in qPCR quantifications we show that their use as housekeeping genes in DNA damage experiments can lead to inaccurate results.

## Introduction

snoRNAs are a well-characterized class of ubiquitously expressed, non-coding RNAs (ncRNAs) that are 60–300 nucleotides in length [1]. Predominantly located in the nucleolus they classically function as guide RNAs for the post-transcriptional maturation and modification of ribosomal RNAs (rRNAs) and snRNAs involved in the spliceosome. snoRNA guide sequences hybridize specifically to their rRNA target sequence, and, via associations with proteins, form small nucleolar ribonucleoprotein complexes (snoRNPs) and execute specific rRNA modifications [1]. Therefore, snoRNAs are crucial for ribosomal function and the effective regulation of translation and thus, unsurprisingly, are highly conserved throughout evolution [2]. There are two major classes of snoRNAs, termed C/D box snoRNAs and H/ACA box snoRNAs, respectively. They differ in terms of their sequence and structure, their binding partners and the nature of the post-transcriptional modifications that they induce [2], [3].

In eukaryotic genomes, snoRNAs are predominantly encoded in the introns of protein-coding host genes but some are under the control of independent promoters [4]. In humans, most snoRNAs are intronic and co-transcribed with their host gene transcripts, and then processed out of the excised introns [5]. However, the transcription of a minority occurs through independent RNA polymerase II or III activity in a similar manner to many miRNAs [5], [6]. Closely related snoRNA family members are usually encoded in different introns of the same host gene, but some host genes encode numerous unrelated snoRNAs. Although some snoRNA host genes appear to be non-protein coding, many are involved in nucleolar function and protein synthesis, and as such there is often an element of co-functioning [5], [7]. The fact that in humans, most snoRNAs are encoded in the introns of protein-coding and non-protein-coding genes gave rise to the assumption that these host genes act solely as cellular housekeepers via their snoRNA-encoding sequences [8], [9]. However, recent studies have challenged this concept and have implicated snoRNAs and their host genes in the control of oncogenesis and cell fate [10], [11]. The existence of a number of ‘orphan’ snoRNAs with no known rRNA targets, and the demonstration of their presence in subcellular locations other than the nucleolus [12], supports the concept that this group of small non-coding RNAs may regulate other molecules and have additional cellular functions [13]. Furthermore, a number of studies suggest an evolutionary relationship between miRNAs and snoRNAs [14] and others report that mature snoRNAs may undergo further cellular processing to form smaller snoRNA-derived RNAs (sdRNAs) with miRNA-like functions [3], [14]–[17]. Additionally, snoRNA expression has been shown to be as variable as miRNA expression in human tumour samples and normalising miRNA polymerase chain reaction (PCR) expression data to these snoRNAs introduced bias in associations between miRNA and outcome [18].

The *growth arrest-specific transcript 5* gene (*GAS5*), located at 1q25, is a non-protein-coding multiple snoRNA host gene comprising of 12 exons [19], [20] initially discovered during screening for potential tumor suppressor genes expressed at high levels during growth arrest. In humans, it encodes ten intronic C/D box snoRNAs and two mature long non-coding RNAs (lncRNAs) isoforms that originate from alternative 5′-splice donor sites in exon 7 [20]. The open reading frame encoded within *GAS5* exons is short and is not thought to encode a functional protein. Mapping of its 5* end demonstrates that it possesses an oligopyrimidine tract characteristic of the 5*-terminal oligopyrimidine (5*TOP) class of genes that accumulate during cell cycle arrest but are rapidly degraded by nonsense-mediated decay during cell growth. The classification of *GAS5* as a 5*TOP gene offers an explanation as to why it is a growth arrest specific transcript as while the spliced *GAS5* RNA is normally associated with ribosomes and rapidly degraded, during arrested cell growth it accumulates in mRNP particles. Interestingly, the only regions of conservation between mouse and human *GAS5* genes are their snoRNAs and 5*-end sequences [20] suggesting that these are the most important functional components. Although *GAS5* plays a role in post-transcriptional modification of ribosomal RNA through its snoRNAs, a number of recent studies have implicated this gene in other important cellular processes [10], [18], [21], [22]. The GAS5 lncRNA was shown to interact with the DNA-binding domain of the glucocorticoid receptor where it acts as a riborepressor, influencing cell survival and metabolic activities during starvation by modulating the transcriptional activity of this receptor [10]. Furthermore, the same group showed in prostate cell lines, that GAS5 mRNA sequesters the androgen/androgen receptor complex and prevents its binding to target DNA sequences [10], which is likely to play an important role in modulating the effects of androgens in the prostate. GAS5 transcripts have also been shown to be important regulators of cell survival and apoptosis in human T-cells and breast and prostate cancer cell lines [21]–[23], and their overexpression sensitized mammalian cancer cell lines to inducers of apoptosis [21]. Furthermore, reduced expression of GAS5 and/or its snoRNAs has been demonstrated in head and neck squamous cell carcinoma [18], breast cancer [18], [21] and glioblastoma multiforme [24], whilst over-expression of U44, U76 and U78 has been shown in NSCLC [25]. The aberrant *GAS5* expression demonstrated in breast and head and neck cancer was associated with poor prognosis [18].

Despite these data, little is known as to the precise role of specific GAS5 snoRNAs in the pathways in which they have been implicated, and even less is accepted about the mechanisms underlying them. Given the previously described role of *GAS5* in the regulation of apoptosis and the well documented role for p53 in the same process, we aimed to further investigate the relationship between p53 and the GAS5 snoRNAs to gain further insight into their potential role in cell survival and oncogenesis in colorectal cancer both *in vivo* and *in vitro*. In addition we demonstrated that both U44 and U47 GAS5 derived snoRNAs, that are amongst the commonest snoRNAs used as housekeeping genes for normalization in association with taqman miRNA expression analysis and should be avoided in DNA damage experiments.

## Materials and Methods

### Ethics Statement

Ethical approval was obtained from Imperial College's ethical review board. The study was performed in accordance with the Declaration of Helsinki and written informed consent was obtained from all patients prior to obtaining tissue samples for the purpose of the study.

### Collection, handling and RNA extraction from laser captured micro-dissected (LCM) tumour samples

**Figure 1:**
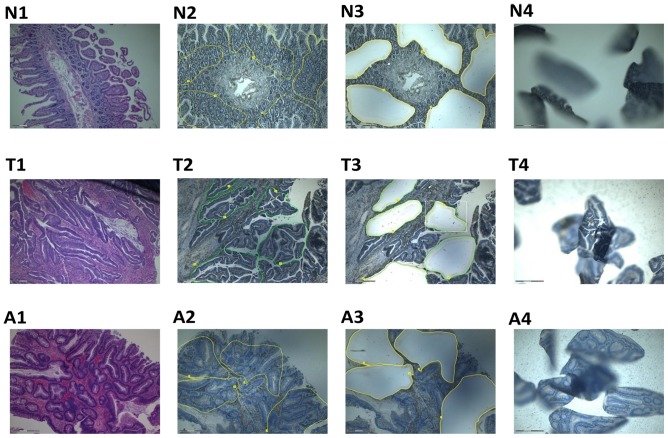
Photographs of tissue blocks on microdissection slides at various stages of the procedure. N = normal colon, T = tumour, A = adenoma. 1: Hematoxylin and eosin staining; 2: areas to be microdissected have been marked; 3: slide following microdissection of marked areas; 4: microdissected tissue following catapulting onto the inside of the eppedORF caps.

With the approval of our institutional review board, tissue specimens representing normal colonic tissue and colonic adenocarcinoma were obtained immediately after surgery, cut into blocks, and then formalin fixed and embedded in paraffin. Written informed consent was obtained. Prior to microdissection, eight 8-µm serial sections were cut (−25°C) from the same tissue block and placed onto slides (1 mm), that were then deparaffinized stained with Hematoxylin and Eosin. They were then microdissected using the PALM Laser MicroBeam system (P.A.L.M. Microlaser Technologies GmbH, Bernried, Germany). A total area of 200,000 µm^2^ was micro-dissected from each slide. Figure 1 shows images of various stages of the microdissection process. RNA was then extracted from the micro-dissected samples the RNeasy MinElute RNA Isolation Kit (Qiagen, Courtaboeuf, France).

### Collection, handling and RNA extraction from macro-dissected colorectal tissue

We wished to investigate whether a relationship existed between p53 activity and the expression of *GAS5*-snoRNAs in human colorectal tissue, and in particular in colorectal tumour samples. We collected paired samples of fresh frozen colorectal tumour tissue and corresponding normal colorectal tissue from 20 individual patients and measured miR-34a and snoRNA levels and p53 expression in these samples. Specimens of normal, adenomatous and malignant colorectal tissue were obtained from individuals undergoing colorectal surgery or colonoscopy between 2011 and 2013 at St Mary's Hospital, London, UK. Written informed consent was obtained from each patient. Samples were immediately macrodissected at the time of surgery, placed directly in RNA*Later* stabilization solution (Qiagen, Hilden, Germany), stored at room temperature for 2–3 hours, and then frozen at −80°C. H&E staining was used for histological confirmation of cancer and to determine the cellularity of representative sections. A specialist colorectal pathologist reviewed the slides, and tissue for RNA isolation was verified to contain ≥60% neoplastic cells. Written informed consent was obtained from all patients, and ethical approval was provided by the hospital's research ethics committee. Fresh tissue stored in RNA*Later* (Qiagen) was crushed in liquid nitrogen and the subsequent powder lysed in Trizol (Invitrogen, Paisley, UK) reagent and RNA isolation was carried out according to manufacturer's instructions.

### Cell culture and doxorubicin treatment

p53 wild-type (WT) and knockout (KO) HCT116 cells and DICER WT and knockdown (KD) cell lines were kindly supplied by Dr. Bert Volgestein [26], [27]. Cells were plated in 150 mm dishes at a 50% confluence and incubated under at 37°C in a humidified 5% CO2 incubator. They were then treated with doxorubicin at a concentration of 0.2 ug/ml or equivalent volume of vehicle (ddh_2_0). After each treatment time point, dishes were placed on ice and medium was aspirated. Cells were washed twice with cold PBS, scraped and centrifuged for 5 minutes at 1300 rpm. The supernatant was removed and the cell pellet was processed for RNA using Trizol reagent (Invitrogen, Paisley, UK) and/or protein extraction.

### RNA quantification and RT-qPCR Analysis

Quantification of extracted RNA was performed using the NanoDrop ND-1000 spectrophotometer (Thermo Scientific, USA) and its quality was analyzed using the RNA 6000 Pico LabChip kit and the 2100 Bioanalyzer (Agilent Technologies, Santa Clara, CA, USA) or via non-denaturing agarose gel electrophoresis. Total RNA (10 ng) was reverse transcribed into cDNA using the TaqMan MicroRNA Reverse Transcription Kit (Applied Biosystems) using a primer specific to each mature miRNA, snoRNA or snRNA. *U6* and *U19* (small nucleolar RNA) was used as endogenous controls for normalization as previously described [28]. qRT-PCR was performed using the TaqMan MicroRNA assaykit (Applied Biosystems). The reaction mix consisted of 10 µl of TaqMan universal master mix 2x, 1 µl of TaqMan mix 20x, 1 ng of cDNA in a final volume of 20 µl. Quantitative real-time PCR (qPCR) was performed with an ABI Prism 7900HT sequence detection system (Applied Biosystems). Data were analysed using qBasePlus software (biogazelle). Levels shown are means of three independent cDNA replications. Normalisation was performed using the delta Ct method.

### SDS-polyacrylamide gel electrophoresis and western blot

Cell pellets or fresh frozen tissue samples were lysed in 30 to 60 µl of NP-40 lysis buffer+protease inhibitors cocktail solution (Roche) and the protein phase was collected after centrifugation. Protein concentration was calculated using the Bradford Reagent Kit (BioRad). Absorbance readings were measured at 595 nm using a Beckman DU 530 Life Science UV/Visible spectrophotometer. After data collection, the concentration of the unknown samples was determined based on standard absorbance value. The protein samples were then exposed to SDS-polyacrilamide gel electrophoresis, transferred to a Hybond C super nitrocellulose membrane (GE Healthcare) and then blotted for the protein of interest. Membranes were washed and Enhanced Chemiluminescence (ECL) detection system (GE Healthcare) was used for visualization. The emitted fluorescence was detected using Hyperfilm ECL (GE Healthcare) on SRX-101A x-ray developer.

### Statistical Analysis

Biostatistical analyses were performed using the GraphPad Prism software. Statistical comparisons were performed using Student's t-test or Pearsons Correlation coefficients.

## Results

### Doxorubicin-induced DNA damage increases *GAS5*-deriveded snoRNA expression in a p53 dependent manner in colorectal cancer cell lines

**Figure 2:**
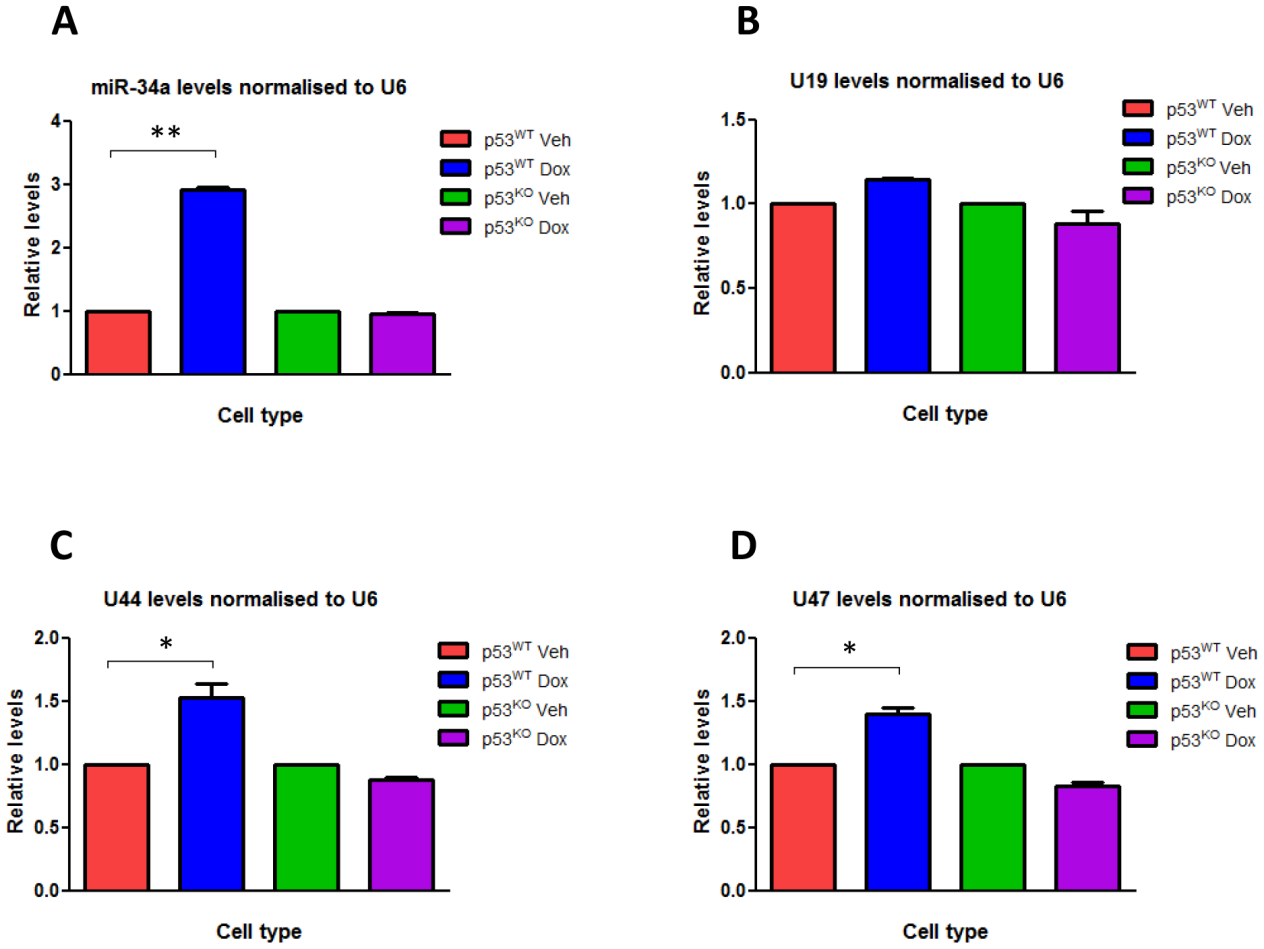
DOX induces the expression of the *GAS5*-derived snoRNAs. Relative levels of (A) miR-34a, (B) U19 snoRNA, (C) U44 snoRNA and (D) U47 snoRNA were measured by RT-qPCR in p53^WT^ HCT116 cell lines and p53^KO^ HCT116 cell lines treated with either doxorubicin (at a final concentration 0.2 ug/ml) or vehicle for 24 hours. Levels were normalised to U6 snRNA levels and data are presented relative to the vehicle treated cells ± s.e.m (each of them performed in triplicate; Student's t test: *P<0.01, **P≤0.006).

**Figure 3:**
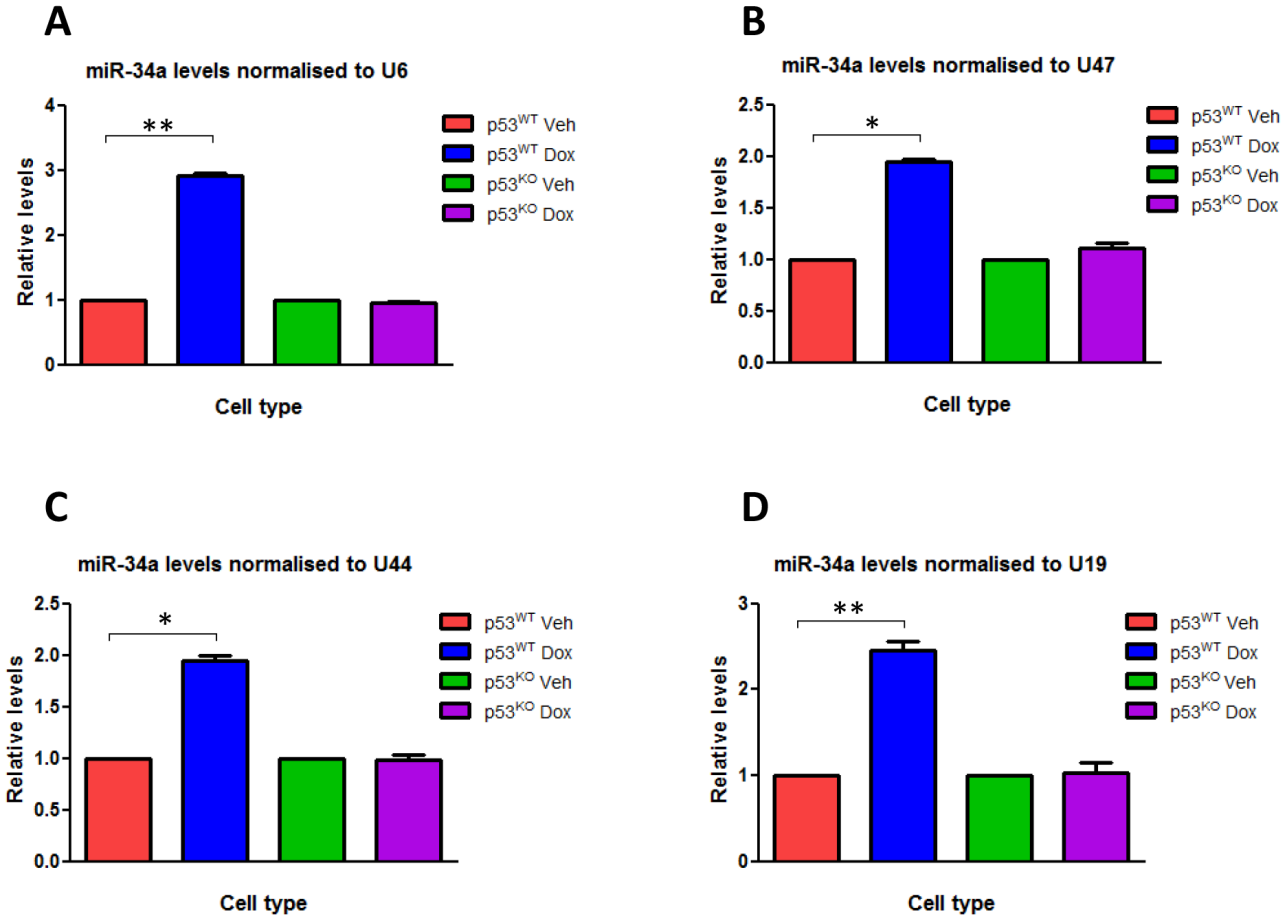
The *GAS5*-derived snoRNAs are not suitable housekeeping genes for use in experiments where DOX treatment. Relative levels of miR-34a normalised to (A) U6 snRNA, (B) U47 snoRNA, (C) U44 snoRNA and (D) U19 snoRNA were measured by RT-qPCR in p53^WT^ HCT116 cell lines and p53^KO^ HCT116 cell lines treated with either doxorubicin (at a final concentration 0.2 ug/ml) or vehicle for 24 hours. Data are presented relative to the vehicle treated cells ± s.e.m (each of them performed in triplicate; Student's t test: *P<0.01, **P≤0.006).

We treated HCT116 p53^WT^ and HCT116 p53^KO^ cells with doxorubicin in order to induce DNA damage, and used RT-qPCR to measure the changes induced in the expression levels of various small RNAs, particularly the *GAS5*-derived snoRNAs U44 and U47. Doxorubicin treatment in HCT116 p53^WT^ cell lines led to a significant induction in the expression of the *GAS5*-derived snoRNAs U44 (P<0.01) and U47 (P<0.01) when compared to treatment with a control vehicle, but there was no significant change in levels of the non-*GAS5*-associated snoRNA U19 (**Figure 2**) or the snRNA U6 that do not derive from the GAS5 locus when compared with GAPDH expression (data not shown). Doxorubicin treatment did not significantly increase *GAS5*-derived snoRNA expression in HCT116 p53^KO^ cells (**Figure 2**), suggesting that DNA damage induced the expression of the *GAS5*-derived snoRNAs in a p53-dependent manner. Doxorubicin treatment of HCT116 p53^WT^ cells also significantly increased the expression of miR-34a (P≤0.006), used as a positive control, although the size of the fold change varied depending on which small RNA was selected to normalize expression levels to (**Figure 2** **&** **3**). Following 24 hours of doxorubicin treatment, miR-34a expression increased significantly by 2.8-fold and 2.6-fold (P≤0.006 for both) when levels were normalised to U6 and U19 respectively. However, although still statistically significant, the fold-changes in miR-34a expression levels were much smaller (1.9-fold; P<0.01) when the *GAS5*-derived snoRNAs U44 and U47 were used for normalisation (**Figure 3**). Similar differences were seen when p21 was used as a positive control (data not shown). Analysis of p53 chromatin immunoprecipitation sequencing (ChIP-seq) experiments performed in HCT116 cells [29] indicates the presence of a significant peak of p53 interaction in two independent experiments involving p53 activation induced by Nutlin3 or 5′fluorouracil (5FU)), at the same position, approximately 800 bp away from the GAS5 transcriptional start site (TSS), indicating that p53 directly controls GAS5 transcription.

### *GAS5*-derived snoRNA expression varies between normal and malignant colorectal fresh non-microdissected tissue in a p53-dependent manner

**Figure 4:**
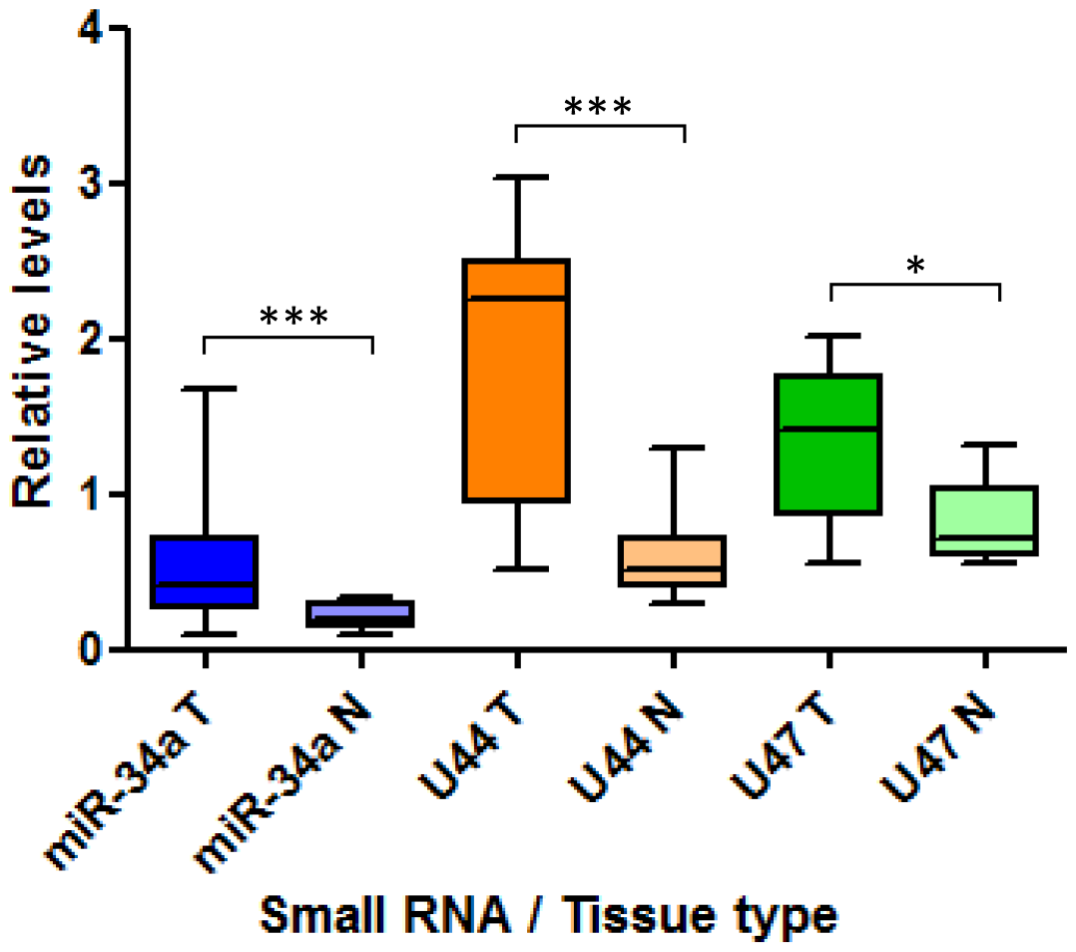
Expression of the *GAS5*-derived snoRNAs is greater in colorectal tumours than in benign colon tissue. A Box plot comparing the relative expression levels of miR-34a, U44 snoRNA and U47 snoRNA between paired colorectal tumour (T) and normal colorectal (N) fresh frozen tissue samples. (Student's t test *P<0.01, ***P≤0.0006).

**Figure 5:**
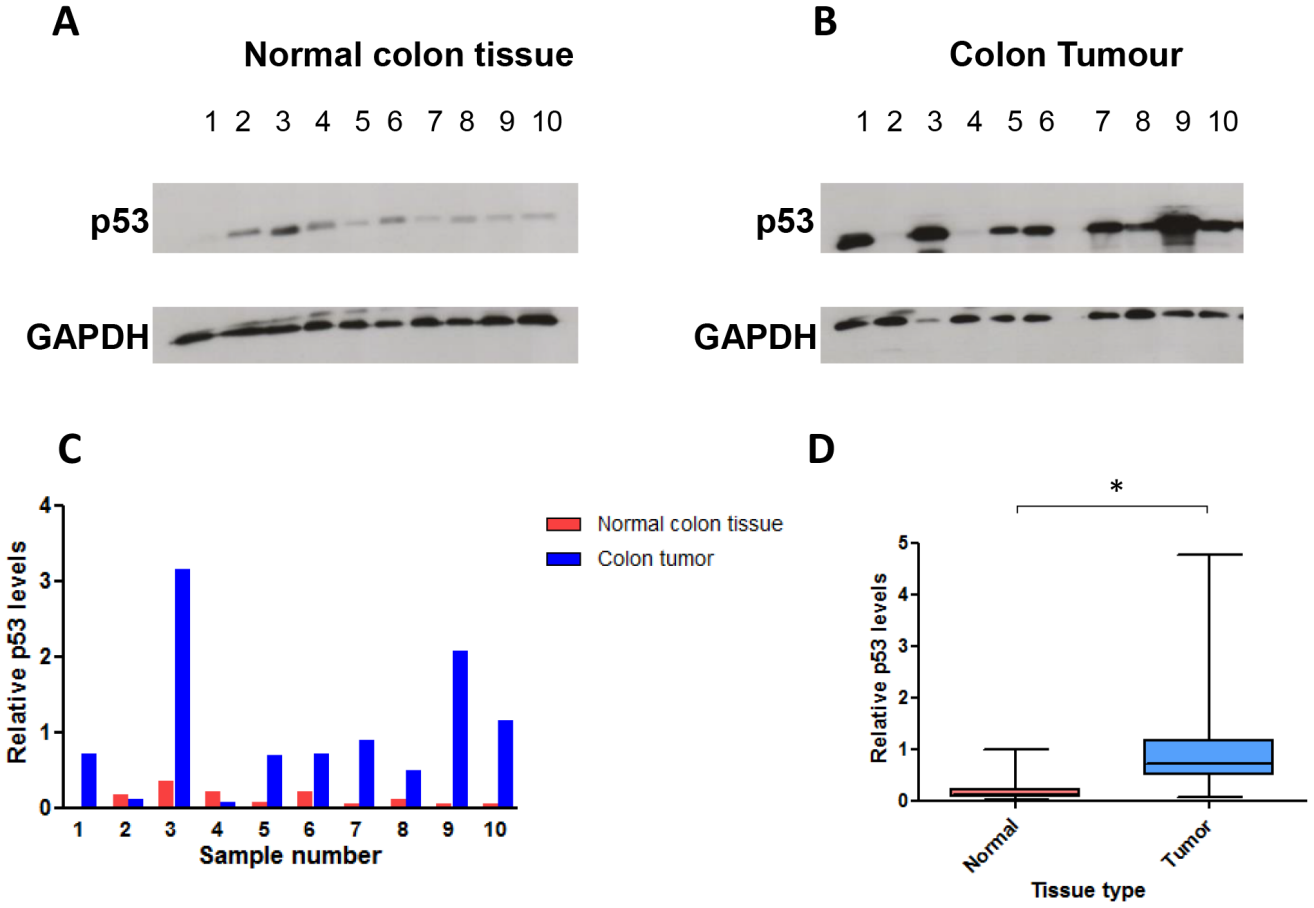
A comparison of p53 expression levels between paired normal colorectal and colorectal tumour tissue samples. (A & B) Western blot's showing p53 levels in the first 10 normal colorectal (A) and colorectal tumour (B) tissue samples. GAPDH was used as a loading control. (C) Column chart demonstrating the fold changes in p53 expression levels shown in the Western blots (A & B) normalised to GAPDH and calculated using ImageJ software. (D) A box plot comparing the relative expression levels of p53 between all 25 paired colorectal tumour (T) and normal colorectal (N) tissue samples (Student's t test *P<0.01).

We found significant differences in the expression levels of the *GAS5*-derived snoRNAs between paired samples of fresh frozen normal colorectal tissue and colorectal tumour from the same patient (P<0.01; **Figure 4**). snoRNA levels were significantly higher in tumours compared to the corresponding normal colorectal tissue in 85% of patient samples, but were significantly lower in 15%. There was no significant difference in snRNA U6 levels between paired normal and tumour samples. Interestingly, miR-34a levels were also significantly higher in patient tumour samples when compared to their corresponding normal colorectal tissue samples (P≤0.0006; **Figure 4**). We then measured p53 expression levels in the paired normal colorectal tissue and colorectal tumour samples using Western blotting (**Figure 5A–C**). p53 levels were significantly higher in colorectal tumours compared to their corresponding normal colorectal tissue samples (P<0.01; **Figure 5D**).

**Figure 6:**
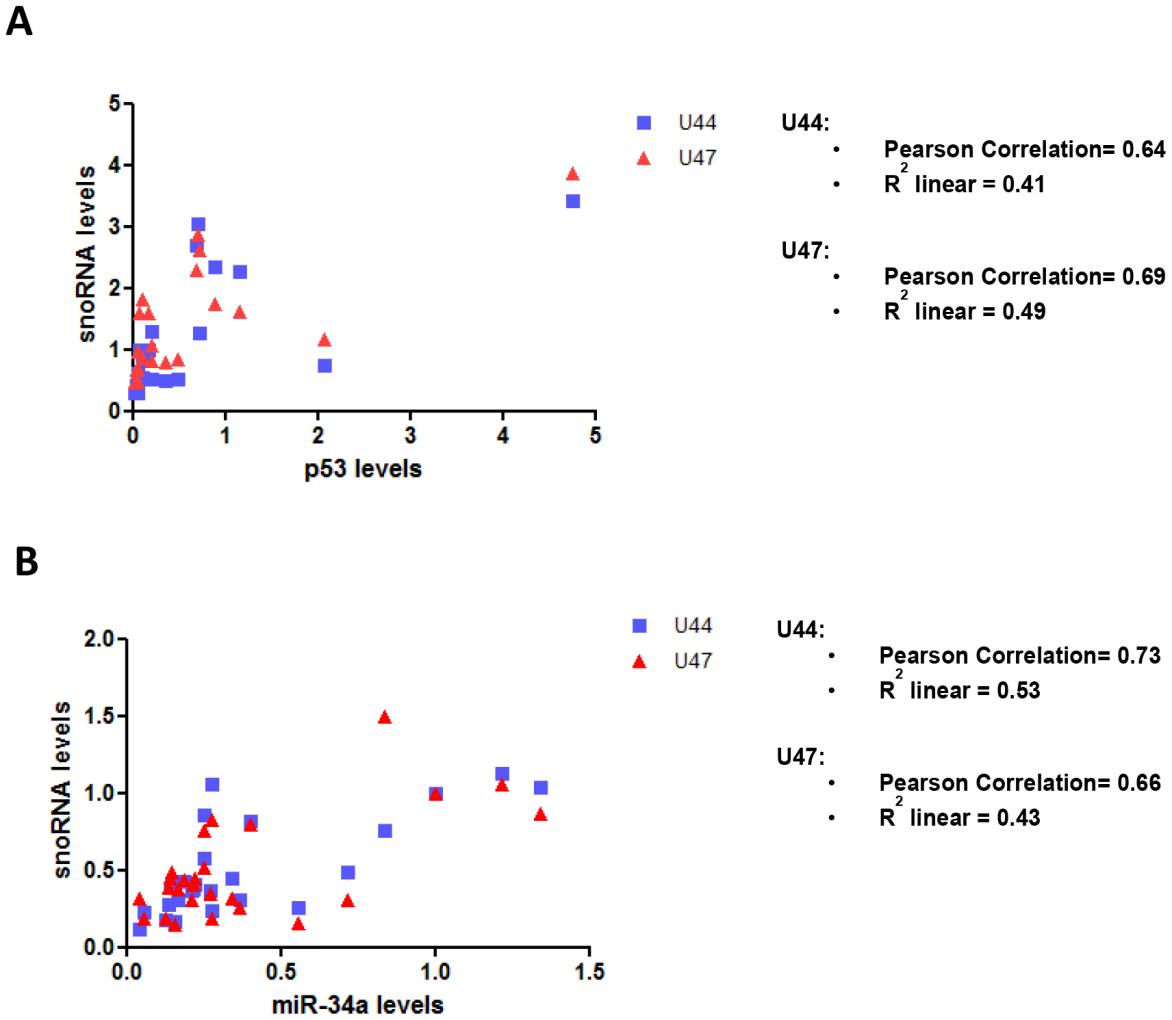
A positive correlation exists between *GAS5*-derived snoRNA levels and p53 expression in colorectal tissue samples. Graphs showing Pearson correlation analyses of the relationship between (A) p53 levels or (B) miR-34a levels and the snoRNAs U44 and U47 in colorectal tumour tissue samples (A & B).

Using the same samples, we calculated Pearson's correlation coefficients, comparing p53 expression levels with snoRNA U44 and U47 levels, to determine if any relationship existed between *GAS5*-derived snoRNA levels and p53 *in vivo* in humans. We found a strong positive correlation between p53 expression levels and the levels of both snoRNA U44 (Pearson Correlation = 0.64; R^2^ linear = 0.41; P = 0.02) and snoRNA U47 (Pearson Correlation = 0.69; R^2^ linear = 0.49; P = 0.01) in colorectal tumour samples (**Figure 6A**). We also calculated Pearson's correlation coefficients to compare miR-34a expression levels with snoRNA U44 and U47 levels, to determine if any relationship existed between the levels of *GAS5*-derived snoRNAs and p53-regulated miRNAs in humans. This was also performed to provide evidence in support of the use of miR-34a as a surrogate marker for p53 in this context for additional experiments using RNA derived from microdissected FFPE tissue samples in which p53 levels were not measurable by Western blotting. Interestingly, we found a strong positive correlation between miR-34a expression levels and the levels of both snoRNA U44 (Pearson Correlation = 0.73; R^2^ linear = 0.53; P = 0.001) and snoRNA U47 (Pearson Correlation = 0.66; R^2^ linear = 0.43; P = 0.02) in colorectal tumour samples (**Figure 6B**).

### *GAS5*-derived snoRNA expression varies between normal, pre-malignant and malignant microdissected FFPE colorectal tissue and levels correlate with miR-34a expression

**Figure 7:**
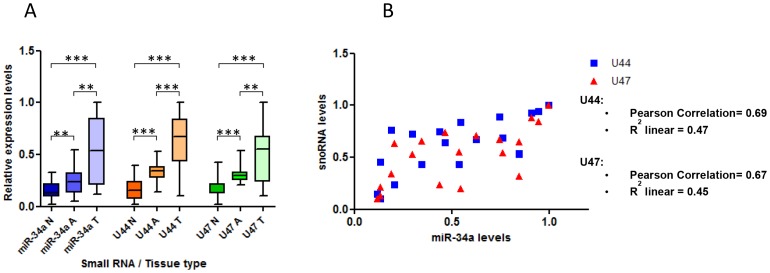
In microdissected colon samples the *GAS5*-derived snoRNAs are expressed more in malignant and pre-malignant tissue than benign tissue and levels correlate with p53 expression. A, RT-qPCR was used to measure the relative expression levels of miR-34a, snoRNA U44 and snoRNA U47 in microdissected human tissue samples corresponding to normal colorectal tissue (N), colorectal adenoma (A) and colorectal tumours (T) (Student's t test *P<0.05,**P≤0.006,***P≤0.0005). B, A Pearson's correlation analysis was performed to determine the relationship between miR-34a levels and the snoRNAs U44 and U47 in microdissected colorectal FFPE tumour tissue samples.

There is much debate as to the accuracy of RNA and gene expression studies that use non-microdissected tumour samples, due to the possible effects that the cellular components of the surrounding stroma can have on the levels of the measured molecule. We therefore aimed to perform further experiments in microdissected tissue samples to support the results above. We collected 60 unpaired FFPE colorectal tissue samples consisting of 20 normal mucosa, 20 adenoma and 20 tumour specimens. We microdissected the required portions after H&E staining, performed RNA extraction and measured small RNA expression levels by RT-qPCR (**Figure 1**). We found significantly higher levels of miR-34a (P≤0.006), snoRNA U44 (P≤0.0005) and snoRNA U47 (P≤0.0005) in adenoma samples when compared with normal mucosa samples (**Figure 7A**). Furthermore, the expression of all 3 small RNAs was significantly higher in tumour samples compared to adenoma or normal mucosa samples (**Figure 7A**). In addition, p53 levels measured by immunohistochemistry and given as a p53 score of 0–3, were higher in tumour samples (80% = score of 3, 20% = score of 2) and adenoma samples (50% = score of 3, 30% score of 2, 20% score of 1) than normal tissue (100% = score of 0).

Using the same samples, we then calculated Pearson's correlation coefficients to compare miR-34a expression levels and snoRNA U44 and U47 levels to determine if the relationship demonstrated in non-microdissected samples between *GAS5*-derived snoRNA levels and p53 (miR-34a being used here as a surrogate marker for p53) was also seen in microdissected colorectal tumours. We found a strong positive correlation between miR-34a expression levels and the levels of both snoRNA U44 (Pearson Correlation = 0.69; R^2^ linear = 0.47) and snoRNA U47 (Pearson Correlation = 0.67; R^2^ linear = 0.45) in colorectal tumour samples (**Figure 7B**).

### The expression of *GAS5*-derived snoRNAs is not affected in colorectal cancer cell lines in which DICER has been knocked-down and therefore do not appear to be processed by DICER

**Figure 8:**
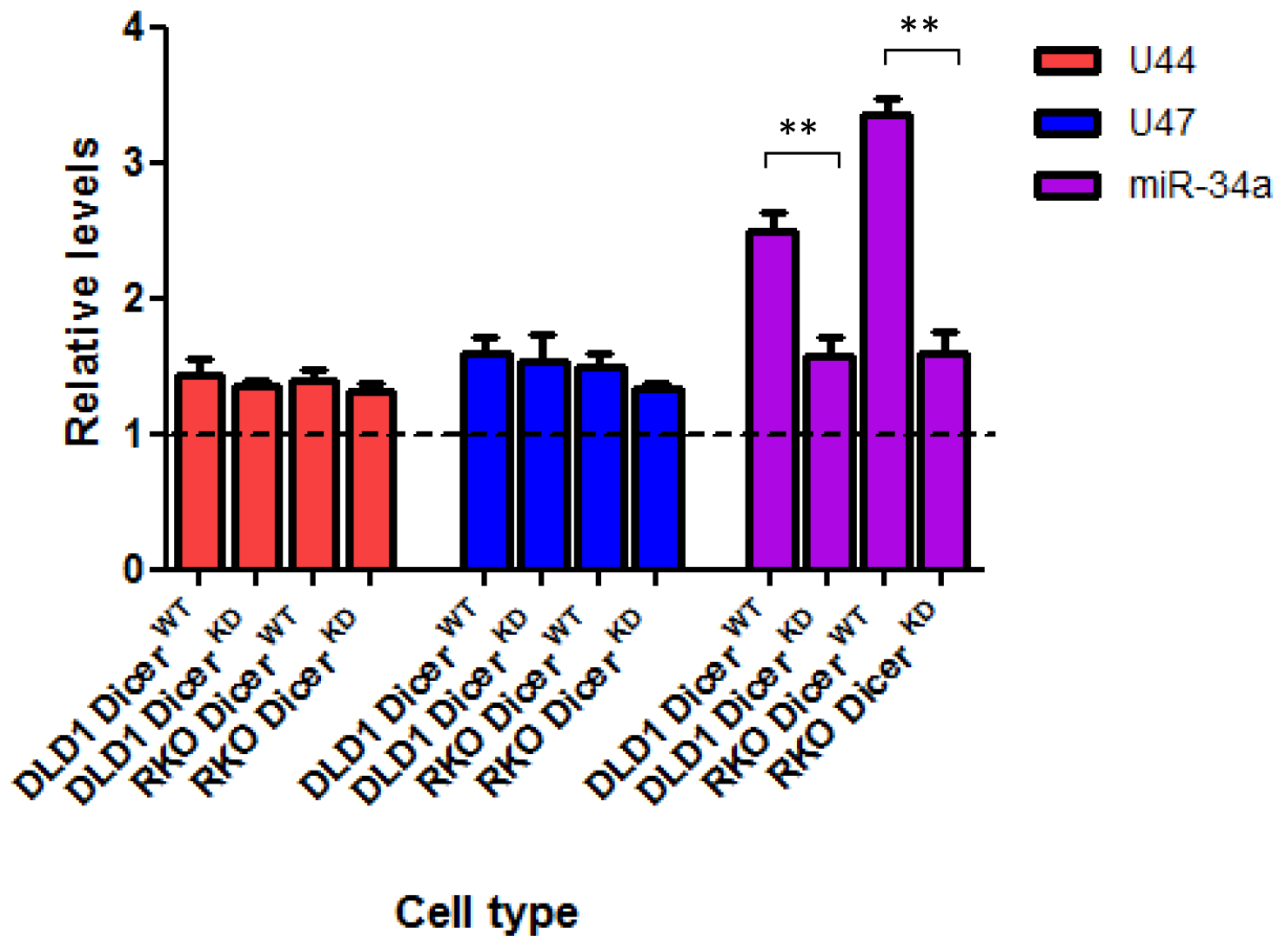
The *GAS5*-derived snoRNAs are not processed by DICER. Relative levels of U44 snoRNA (red), U47 snoRNA (blue) and miR-34a (purple) were measured by RT-qPCR in DLD1 DICER^WT^ cell lines, DLD1 DICER^KD^ cell lines, RKO DICER^WT^ cell lines, RKO DICER^KD^ cell lines treated with either doxorubicin (at a final concentration 0.2 ug/ml) or vehicle for 24 hours. Data are presented relative to the vehicle treated corresponding cell lines (dotted line) ± s.e.m (each of them performed in triplicate; Student's t test: *P<0.01, **P≤0.006).

In view of the findings described in previous studies which demonstrated that snoRNAs can be converted by DICER to sdRNAs with miRNA-like functions it is possible that miRNA-like molecules produced from GAS5 derived snoRNAs are involved in the DNA damage response [3]. To investigate the possible involvement of DICER in this process we assessed the effect of DICER knock-down on the expression of *GAS5*-derived snoRNAs following DNA damage. To achieve this we used RT-qPCR to compare changes in the expression of the snoRNAs U44 and U47 following doxorubicin treatment in the colorectal cancer cell lines DLD1 and RKO in their wild-type (WT) form and in a form in which DICER had been stably knocked down (KD). Interestingly, we found that although DICER knock-down led to a statistically significant reduction in miR-34a levels (P≤0.006) in both DLD1 and RKO cell lines, as expected, there was no effect on the levels of snoRNA U44 or snoRNA U47 (**Figure 8**), suggesting that the function of the *GAS5*-derived snoRNAs in the p53-regulated response to DNA damage did not involve their conversion into sdRNAs with miRNA-like function.

## Discussion

Our findings demonstrated a relationship between p53 activity and the expression of the *GAS5*-derived snoRNAs in colorectal cancer cell lines and human colorectal tissue. Furthermore, they suggested that transcription of the *GAS5* gene is directly regulated by p53, although chromatin immunoprecipitation studies would be required to confirm this. Although no functional studies have been performed, these findings suggested an important role for the *GAS5*-derived snoRNAs in the p53-regulated cellular response to DNA damage and in p53-associated signalling pathways in human colorectal tissue and colorectal cancer.

Until Chang *et al.* (2002) [30] first described the potential role of snoRNAs in tumorigenesis, there had been little justification for the systematic evaluation of the role of snoRNAs in this or any other pathological condition. However data are accruing that link a dysregulation in the expression of various snoRNAs to the development of a number of malignancies [11], [25], [31], [32]. As the *GAS5* gene hosts ten intronic snoRNAs and a lncRNA and has been implicated in oncogenesis and the regulation of cell survival and apoptosis [21]–[23], and given the well documented role for p53 in the same processes, we aimed to further investigate the relationship between p53 and the GAS5 snoRNAs to gain further insight into their potential role in tumorigenesis. We found that, in colorectal cancer cell lines, the *GAS5*-derived snoRNAs were induced in a p53-dependent manner following DOX stimulated DNA damage but that this affect was not lost when DICER was functionally knocked down. This suggested that these snoRNAs were not processed into sdRNAs with miRNA-like function, and that their role in the DNA damage response did not require them to be further processed in this manner. This indicates that these snoRNAs might be involved in coordinating the p53-mediated response through their role in regulating the ribosome. snoRNAs are crucial for ribosomal function and the effective regulation of translation [2] and p53 is a key mediator of ribosome biogenesis especially in response to so-called nucleolar stress [33]. Furthermore, p53 has been shown to mediate the signaling link between ribosome biogenesis and the cell cycle [34]. It seems logical therefore that the *GAS5*-derived snoRNAs might be directly induced by p53-mediated transcription following DNA damage in order to ‘streamline’ the post-transcriptional maturation and modification of rRNAs and to ensure a more efficient translation of genes required to coordinate a response to such a stress. This theory clearly requires further experimental evaluation not least by proving that there is an increase in the localization of these snoRNAs to the ribosome rather than an alternative cellular compartment following DNA damage. It is possible that these snoRNAs do act at a location other than the ribosome and that they may have sdRNA type function but do not require DICER processing to enable this. Whether these DNA damage induced *GAS5*-derived snoRNAs simply function to accommodate an increase in gene translation at the ribosome or whether they are indeed processed to sdRNAs and have additional, independent function, their effect on gene expression could be assessed through over-expression experiments followed by gene profiling experiments. This would enable us to determine their relevance in terms of facilitating the p53-dependent response to DNA damage through the post-transcriptional regulation of gene expression.

Following on from the cell line experiments, we further demonstrated that a strong correlation existed between p53 levels and *GAS5*-derived snoRNA expression in normal, pre-malignant and malignant human tissue samples and that this might have relevance in tumorigenesis. We found a strongly positive correlation between p53 and the *GAS5*-derived snoRNAs in all three tissue types and in both the micro-dissected FFPE samples (where miR-34a was used as a surrogate marker for p53) and the fresh non-micro-dissected samples. This provided further evidence in support of a role for p53 in the induction of the *GAS5*-derived snoRNAs and suggested a function for this process *in vivo*. Interestingly U44 and U47 levels were found to be higher in tumour samples than in normal or pre-malignant tissue which is most likely the result of higher p53 expression in these tumours. Remarkably, and some would argue paradoxically, p53 is overexpressed in up to 50% of colorectal cancers and this has been associated with a favourable prognosis in some studies [35], [36]. This paradox may be explained by the fact that in many cases it is mutant rather than wild-type p53 that is overexpressed and hence the effect on cell phenotype will vary based on the function of the mutation variant [36]–[38]. On the other hand p53 is induced by oncogene expression as well as by DNA damage, so it is possible that an oncogenic pathway that is driving that particular tumour also leads to p53 induction. In fact, the survival for patients with colorectal cancers expressing mutated p53, has been shown to be significantly worse than for those patients with tumours that expressed the wild-type protein and this was most striking when mutations occurred outside the evolutionarily conserved regions [38], [39]. We did not specifically differentiate between mutant and wild-type p53 expression in our study and it would be interesting to investigate in future work whether the same correlation exists between mutant p53 and the *GAS5*-derived snoRNAs in human tumour samples. It is likely that this will be dependent on the specific p53 mutation as many do retain transcriptional activity although often the gene set varies from that regulated by wild-type p53. Interestingly, in our cell line work we saw an induction in *GAS5*-derived snoRNA expression following DOX treatment in the DLD1 cells which are known to contain the R241F p53 mutant, and this demonstrated that this mutant form of the protein was capable of transcriptionally activating the *GAS5* gene. It would also have been interesting to correlate *GAS5*-derived snoRNA levels with outcome in these patients, however as these samples were all taken from patients with resectable Dukes Stage A–C tumours there has not been a significant gap since their diagnosis to accrue statistically meaningful results as the data are too immature.

Finally, our experiments showed that the *GAS5*-derived snoRNAs were not appropriate to be used as housekeeping genes for normalising RT-qPCR experiments that used DOX to induce DNA damage. We found that the relative expression of p53-regulated miRNAs significantly differed depending on whether *GAS5*-derived snoRNAs or alternative housekeeping genes such as U6 snRNA or U19 snoRNA were used to normalise results. This implies that the use of the *GAS5*-derived snoRNAs as normalising genes in the context of DNA damage experiments would lead to an inaccurate interpretation of the results, and suggests that the snRNA U6 or snoRNA U19 would be more appropriate for normalisation in such experiments.This is in keeping with the findings of others who have shown that in experiments involving human tumour samples, snoRNA expression was as variable as miRNA expression and that normalising miRNA PCR expression data to these snoRNAs introduced bias in associations between miRNAs and outcome [18].
